# Parenteral Nutrition Containing Fish Oil for Hospitalized Non-Intensive Care Unit (ICU) Patients: A Systematic Review, Meta-Analysis, and Cost-Effectiveness Analysis

**DOI:** 10.3390/nu17071284

**Published:** 2025-04-07

**Authors:** Lorenzo Pradelli, Axel Rüdiger Heller, Stanislaw Klek, Konstantin Mayer, Martin D. Rosenthal, Maurizio Muscaritoli

**Affiliations:** 1 AdRes-Health Economics and Outcome Research, Via Vittorio Alfieri 17, 10121 Turin, Italy; 2 Department of Anaesthesiology and Intensive Care Medicine, University of Augsburg, 86159 Augsburg, Germany; axel.heller@uk-augsburg.de; 3 Surgical Oncology Clinic, The Maria Sklodowska-Curie National Cancer Institute, 31-115 Krakow, Poland; klek@poczta.onet.pl; 4 Department of Internal Medicine, University Hospital of Giessen and Marburg, Klinikstraße 33, 35392 Giessen, Germany; konstantin.mayer@innere.med.uni-giessen.de; 5 Department of Surgery, Division of Trauma and Acute Care Surgery, University of Florida College of Medicine, Gainesville, FL 32610, USA; martin.rosenthal@surgery.ufl.edu; 6 Department of Clinical Medicine, Sapienza University of Rome, 00185 Rome, Italy; maurizio.muscaritoli@uniroma1.it

**Keywords:** cost-effectiveness, fish oil, lipid emulsion, meta-analysis, omega-3, parenteral nutrition

## Abstract

**Background and aims**: We investigated parenteral nutrition (PN) containing fish oil (FO-PN) vs. standard PN without fish oil (NF-PN) in adult patients hospitalized in the non-intensive care unit (general ward). **Methods**: Searches in Medline, Embase, and Web of Science (any date to 10 October 2024) were screened, data were extracted, and the quality of the studies was assessed by two independent researchers. Meta-analyses were performed, with outcomes included in random effects models, and heterogeneity for clinical outcomes was explored via subgroup analyses and meta-regression. Pharmacoeconomic analyses used data from the current meta-analysis. **Results**: In this study, 29 randomized controlled trials (RCTs) were included, with intervention and control groups given FO-PN and NF-PN, respectively, as part of PN covering ≥70% energy provision. Compared to NF-PN, FO-PN was associated with a 37% lower relative risk (RR) of infection (19 RCTs; RR 0.63, 95% confidence interval [CI] 0.50–0.78; *p* < 0.0001), 2.03 days shorter length of hospital stay (18 RCTs; 95% CI 1.23–2.84; *p* < 0.00001), and a 51% reduction in the risk of sepsis (10 RCTs; RR 0.49, 95% CI 0.32–0.74; *p* = 0.0009). There was a non-significant 54% reduction in the 30-day mortality rate (11 RCTs; RR 0.46, 95% CI 0.20–1.08; *p* = 0.07) for FO-PN. FO-PN was associated with better clinical outcomes and financial savings (i.e., dominance) compared to NF-PN in all five countries studied. **Conclusions**: FO-PN is a cost-effective option compared to NF-PN for adult patients hospitalized in a general ward across a range of healthcare systems.

## 1. Introduction

Malnutrition in hospitalized patients remains common and is both under-identified and under-treated [1]. Malnutrition can be associated with poorer outcomes, including an increased incidence of infectious and non-infectious complications and in-hospital mortality, and result in greater healthcare utilization (e.g., longer hospital or intensive care unit (ICU) stays, hospital (re)admissions, and increased medication use) [2]. Parenteral nutrition (PN) is useful for addressing malnutrition when oral or enteral nutrition is not possible, is insufficient, or is contraindicated, and it includes lipid emulsions as an essential component [3,4,5]. The lipid source in PN is also important. Early ‘first-generation’ lipid emulsions, used as part of PN, were based solely on soybean oil or soybean/safflower oil, providing high levels of omega-6 polyunsaturated fatty acids (PUFAs) [6,7]. Following concerns that the relatively high omega-6 PUFA concentration supplied by soybean oil lipid emulsions might be inflammatory and immunosuppressive, more complex composite blends were developed using mixed-oil sources, including medium-chain triglycerides (MCTs), olive oil, and fish oil [7,8,9,10].

Modern composite lipid PN emulsions frequently contain fish oil [10]. In part, this is because of accumulated evidence concerning the biological effects of fish oil, attributed principally to the omega-3 PUFAs, docosahexaenoic acid (DHA), and eicosapentaenoic acid (EPA) [10,11]. As well as having anti-inflammatory, immunomodulatory, and anti-oxidative effects, DHA and EPA are precursors of specialized pro-resolution mediators (i.e., resolvins, protectins, and maresins) that have been shown to exert potent beneficial effects in many animal disease models, including immune modulation and tissue repair [10,12]. Moreover, EPA and DHA may help preserve muscle mass and strength, which are important determinants of patient recovery following surgery, periods of immobility, or critical illness [13,14]. Thus, there is a firm biological rationale for the inclusion of fish oil in PN; so, numerous clinical trials have compared PN with and without fish oil in a variety of clinical conditions and patient groups.

The current analysis of adult hospitalized patients given PN focuses on hospitalized patients treated in a general ward, thus excluding ICU patients. Compared to those in the ICU, general ward patients requiring PN are a somewhat heterogeneous group, united by a need for PN in order to reach their energy requirements, although the majority are surgical patients with intestinal failure from a variety of causes [5,15]. PN for surgical patients may be most beneficial in undernourished patients when enteral nutrition is not feasible or not tolerated and in those with postoperative complications impairing gastrointestinal function who are unable to receive and/or absorb adequate oral/enteral nutrition for at least seven days [16]. In contrast with ICU patients requiring PN, general ward patients tend to be in a non-hypercatabolic state; thus, their protein and energy targets generally tend to be less than those of hypercatabolic patients.

We previously conducted a systematic review and meta-analyses that demonstrated notable clinical advantages of FO-PN in adult hospitalized patients, including a 40% and 56% reduction in the risks of infection and sepsis, respectively, as well as a decrease of approximately two days in both ICU and overall hospital stay [17]. As ICU and general ward patients formed distinct subgroups within this meta-analysis, a follow-up study further analyzed the ICU sub-population with regard to PN with and without fish oil [18]. The current study now seeks to investigate an updated general ward patient sub-population in greater detail as the initial analysis [17] did not allow clear conclusions to be drawn for the general ward patient population as they were grouped together with those in the ICU. Furthermore, general ward adult patients requiring PN represent a numerous and important group, comprising more than half of all patients in the initial meta-analysis that included both ICU and general ward patients [17]. Thus, the objective of this study was to perform a systematic review and meta-analysis, as well as a cost-effectiveness analysis, for adult patients hospitalized in a general ward, investigating the potential benefits of FO-PN vs. NF-PN.

## 2. Methods

### 2.1. Meta-Analysis

*Registration and overview*. This research followed current best practices, such as the prospective registration of methods with the international prospective register of systematic reviews (PROSPERO: PROSPERO 2021 CRD42021293972) [19] and adhering to the preferred reporting items for systematic reviews and meta-analysis (PRISMA) statement for reporting systematic reviews and meta-analyses [20]. Prospectively identified outcomes from included studies were extracted, pooled, and meta-analyzed according to the Cochrane Handbook for Systematic Reviews of Interventions [21]. This study concerns the assessment of clinical efficacy and safety in adult patients hospitalized in a general ward given FO-PN or NF-PN and also investigates how results vary by the type of comparator and patient characteristics. The methods included (a) setting the eligibility criteria, (b) identifying databases and formulating the search strategy, (c) conducting a structured literature search followed by the stepwise screening of titles, abstracts, and full texts, (d) extracting data and synthesizing results for the meta-analysis, and (e) performing a cost-effectiveness evaluation.

#### 2.1.1. Eligibility Criteria

Study eligibility was defined using the PICOS framework (participants, interventions, comparisons, outcomes, and study designs) [21,22]. The included studies involved adult hospitalized patients outside the ICU (i.e., in medical or surgical wards) who received PN providing at least 70% of total energy requirements. Studies involving pediatric, neonatal, or ICU populations or those focused on enteral nutrition were excluded. (Note: for the purpose of subgroup analysis, ‘total PN’ studies were defined as those excluding any use of enteral and/or oral nutrition, whereas for ‘PN studies’, enteral and/or oral nutrition could contribute up to 30% of the total calories supplied.) FO-PN formed the intervention group, and NF-PN was the control group. Studies were excluded if they involved ‘off-label’ PN use (e.g., fish oil as the sole parenteral lipid source) or if enteral nutrition accounted for over 30% of daily energy intake. The co-primary clinical endpoints were infection rates and 30-day mortality. (In this analysis, 30-day mortality referred to deaths occurring within 30 days of receiving at least one dose of the intervention or before hospital discharge, depending on the data reported). The secondary main clinical outcomes were length of hospital stay, sepsis rate, and hospital readmissions. Additional outcomes included plasma phospholipid fatty acid composition and lipid parameters (α-tocopherol, EPA, DHA, and plasma triglyceride levels); inflammatory and antioxidant markers (changes in interleukin-6, leukotrienes B4 and B5, LTB5:LTB4 ratio, C-reactive protein, and TNF-α); and standard laboratory values such as urea, serum creatinine, platelet count, liver enzymes (ALT, AST, and GGT), and both total and direct bilirubin. The analysis was restricted to randomized controlled trials (RCTs) published in English in peer-reviewed journals that reported at least one pre-specified outcome.

#### 2.1.2. Information Sources and Search Methods

A structured search strategy was formulated *a priori* using the PICOS criteria [19,21,22], and the search keywords were “parenteral nutrition”, “fish oil”, “lipids”, “emulsion”, and “randomised controlled trial”. No restrictions or filters were used, with an inclusion time interval prior to 30 September 2024. Medline (PubMed interface), Embase, and Web of Science were searched using search strings modified to fit each database’s requirements (Table S1). The results were combined to create a core database, eliminating duplicate records. Manual searches of included study reference lists were performed, along with reviews and meta-analyses on the subject, and any additional RCTs identified were added to the core database.

#### 2.1.3. Study Selection, Data Collection, Summary Measures, and Individual Study Bias Assessment

Screening of core database publications by inclusion and exclusion criteria was performed independently by two authors, first looking at titles and abstracts and then at the full text of eligible papers. Conflicting opinions were resolved by consulting with a third review author. Data were extracted from included studies by two authors working independently using a predefined standardized collection grid, with disagreements addressed through consultation with the principal investigator. Outcomes shown only as figures were converted to numerical values using Engauge^®^ software, version 12.1 [23]. Standard errors of the mean (SEMs) were converted to standard deviations (SDs) using established equations, while medians and interquartile ranges were transformed into estimated means and SDs following the method proposed by Wan et al. [24]. Continuous outcomes were summarized using the weighted mean difference with 95% confidence intervals (CIs) or the standardized mean difference when varying measurement scales were present. For dichotomous outcomes, risk ratios (RRs) with a 95% CI were calculated. Two authors independently evaluated the risk of bias in each study using the Cochrane Collaboration’s bias assessment tool [21]. Bias was judged as a graded set of response options (from ‘low’ to ‘some concerns’ to ‘high’). (Note: the prospectively defined methods [PROSPERO 2021 CRD42021293972] involved assessing the risk of bias according to the Risk of Bias [RoB] 1.0 tool, but in the current analysis, RoB 2.0 was used).

#### 2.1.4. Synthesis of Results

Meta-analyses were conducted using Review Manager (RevMan 5.4), developed by the Nordic Cochrane Centre for the Cochrane Collaboration when studies were sufficiently homogeneous in terms of design and comparator. All outcomes were analyzed using random effects models. In cases of substantial heterogeneity (*I*^2^ > 50%), potential sources were investigated through subgroup analyses and meta-regression, with stratification based on patient profiles, intervention type, study features, and clinical context (i.e., age, sex, reason for PN, nutrition status, oncological setting, and PN/TPN), provided ≥5 studies reported on it.

### 2.2. Pharmacoeconomic Analysis

The pharmacoeconomic and associated sensitivity analyses were performed essentially as detailed previously for an ICU patient population [18]. In brief, this consisted of cost-effectiveness models using data from the current meta-analysis. Five cost-effectiveness models were based on a probabilistic discrete event simulation technique run over 10,000 iterations, developed and simulated for hospitals in France, Germany, Italy, Spain, and the UK, to compare FO-PN vs. NF-PN in adult patients hospitalized in a general ward. Studies included in the meta-analysis were used to value the weighted means for hospital length of stay and infection rate (weights based on patient number for the NF-PN arm of each study), and outcomes for the FO-PN group were simulated after applying relative efficacy estimates from the meta-analyses to the outcomes of the NF-PN group. Death rates were set as equal in both arms given the inconclusive results for this parameter in the meta-analysis. Economic parameters such as daily ward and infection costs are country-specific and have been reported previously [18]. Probabilistic sensitivity analyses were performed by drawing parameter values from their respective probability distributions, creating 1000 unique sets of parameter combinations. If data concerning uncertainty were missing, a 20% standard deviation of the mean value was used with an appropriate probability distribution. For deterministic sensitivity analyses, simulations were repeated while varying parameter values to their upper and lower CI limits and keeping other parameter values constant. Where CIs were unavailable, the lower and upper 95% CIs of the distribution used in probabilistic sensitivity analyses were assumed as parameter values.

## 3. Results

### 3.1. Study Selection and Characteristics

**Figure 1:**
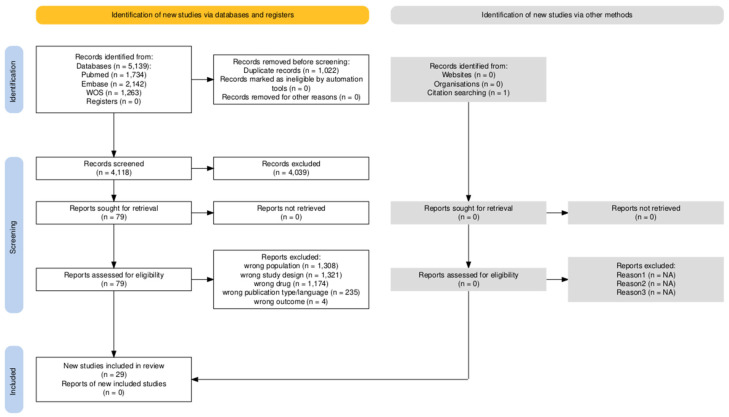
Study selection and screening.

Data from 2587 patients enrolled in 29 randomized controlled trials were incorporated into the systematic review and meta-analytic synthesis (Figure 1 and Table 1) [25,26,27,28,29,30,31,32,33,34,35,36,37,38,39,40,41,42,43,44,45,46,47,48,49,50,51,52,53]. Risk-of-bias results are presented in Figure S1.

### 3.2. Clinical Outcomes

**Figure 2:**
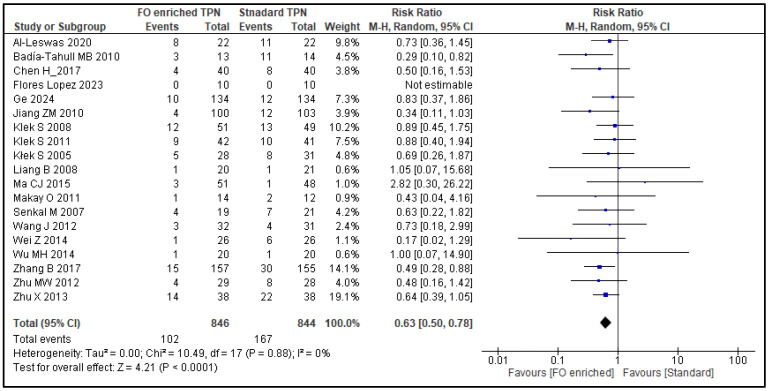
Infection rates: forest plot of a meta-analysis of random effects, showing individual study means and pooled estimates. [26,27,28,30,31,34,35,36,37,39,42,43,46,47,48,49,50,52,53].

**Figure 3:**
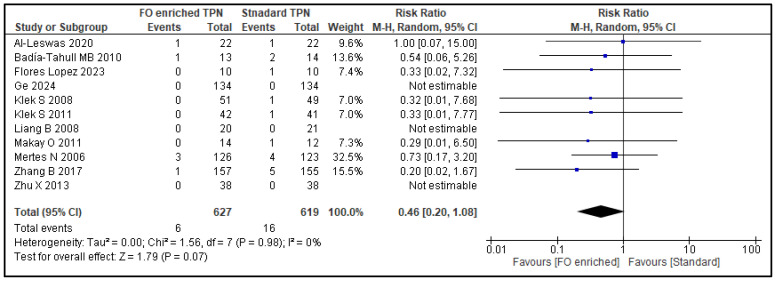
Overview of 30-day mortality rates: forest plot from a meta-analysis of random effects, displaying individual study estimates and the overall pooled effect. [26,27,30,31,36,37,39,43,44,50,53].

A total of 19 studies (1690 patients) reported nosocomial infections and were assessed for the co-primary outcome, infection rate. Compared to NF-PN, FO-PN resulted in a 37% reduction in infection rates (RR 0.63, 95% CI 0.50–0.78; *p* < 0.0001) (Figure 2). Subgroup analysis was not performed as heterogeneity was low (*I*^2^: 0%). Eleven studies (1246 patients) reported the co-primary outcome, namely, mortality rate. There was a non-significant 54% reduction trend in the 30-day mortality rate for FO-PN (RR 0.46, 95% CI 0.20–1.08; *p* = 0.07) (Figure 3). Again, subgroup analysis was not performed due to low heterogeneity (*I*^2^: 0%).

**Figure 4:**
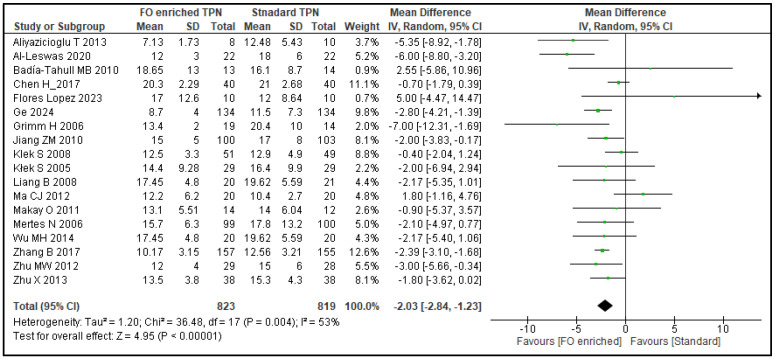
Length of hospital stay: forest plot of a meta-analysis of random effects, showing individual study means and pooled estimates. [25,26,27,28,30,31,32,34,35,36,39,41,43,44,49,50,52,53].

Length of hospital stay reports were found for 18 studies (1642 patients). The results showed a reduction in the length of hospital stay of 2.03 days (95% CI 1.23–2.84; *p* < 0.00001) with FO-PN (Figure 4). As the data for length of stay outcomes were classified as highly heterogeneous (*I*^2^ = 53%), subgroup analyses and meta-regression were performed. The subgroup analyses showed a significantly greater reduction in length of hospital stay for 8 total PN studies (680 patients) including fish oil (3.46 days; 95% CI 2.09–4.83) than for 10 PN studies (962 patients) including fish oil (1.29 days; 95% CI 0.46–2.13), with a significant (*p* = 0.008) test for differences between groups. (Note that ‘total PN’ studies were defined as those excluding any use of enteral and/or oral nutrition, whereas in ‘PN studies’, enteral and/or oral nutrition could contribute up to 30% of total calories supplied.) Thus, total PN may be associated with the observed reduction in the length of hospital stays for patients receiving omega-3 fatty acids. A similar subgroup analysis by oncological status did not reveal a significant difference (*p* = 0.4), meaning that the observed reduction in length of hospital stay might not be associated with the presence of an oncological diagnosis. Likewise, no association was observed when analyzing malnourished vs. non-malnourished subgroups (*p* = 0.75). Meta-regression for all predefined covariates showed that the best model describing heterogeneity includes both the proportion of males and total PN as these two covariates explain almost two-thirds of the between-study variance, with the *I*^2^ reducing from 53% to 30%. Residual unexplained heterogeneity may relate to the differences in patient characteristics and study procedures.

**Figure 5:**
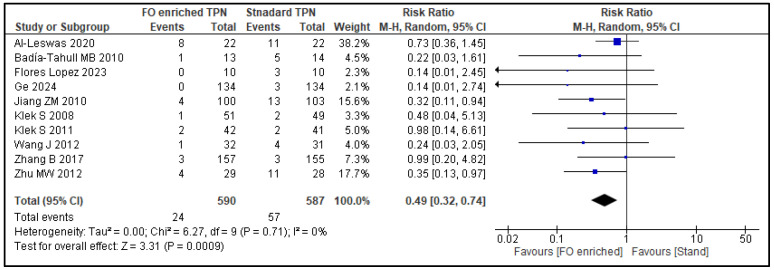
Sepsis: forest plot of a meta-analysis of random effects, showing individual study means and pooled estimates. [25,26,27,30,31,34,36,37,47,50,52].

Out of the 29 included studies, 10 (1117 patients) distinguished between infection rate and the occurrence of sepsis, reporting both percentages independently. Compared to NF-PN, FO-PN significantly reduced the risk of sepsis by 51% (RR 0.49, 95% CI 0.32–0.74; *p* = 0.0009) (Figure 5). No subgroup analysis was performed as heterogeneity was low (*I*^2^: 0%). Meta-analyses were not performed on hospital readmissions as only one study reported this outcome.

### 3.3. Non-Clinical Outcomes

Of the 19 laboratory parameters assessed (Table S2), significant improvements were observed in 9 of them. These included reductions in liver enzyme markers (AST, ALT, and GGT), elevated antioxidant levels (α-tocopherol), and a decrease in inflammatory markers such as TNF-α. Fatty acid profiles improved, with increased concentrations of omega-3 fatty acids, DHA, and EPA. Additionally, a beneficial effect on leukotriene levels was noted, marked by a rise in LTB5 and an enhanced LTB5:LTB4 ratio.

### 3.4. Pharmacoeconomic Analyses

Model input data derived from clinical results were used for all five countries. These were a mean reduction of 1.99 days for length of hospital stay and a mean reduction of 841 infections per 10,000 patients, both in favor of FO-PN. Cost results and the cost-effectiveness analysis for the 10,000 patient-level simulations conducted for each country are shown in Table 2, and sensitivity analyses are also presented (Figures S2–S6). Taken together, these pharmacoeconomic results show that the use of FO-PN was associated with better expected clinical outcomes *and* concurrent savings (i.e., dominance) in the base case and all sensitivity tests conducted compared to standard PN.

## 4. Discussion

The current study indicates that FO-PN significantly reduces the risk of infections, sepsis, and length of hospital stays compared to standard PN in hospitalized general ward patients given PN to cover at least 70% of their nutritional needs. Furthermore, FO-PN has potential beneficial effects on marker liver enzyme levels, antioxidant status, and markers of inflammation and improves the fatty acid profile. The clinical outcomes can be compared to our previously published meta-analyses concerning FO-PN vs. NF-PN in the overall group of all hospitalized patients (ICU and general ward populations) [17], as well as the subgroup of ICU patients (Table S3) [18]. All three studies show similar (about 40%) significant reductions in infections and hospital length of stay (between 2 and 3 days). A significant (51%) reduction in sepsis is observed in the current study, as well as in the study including all hospitalized patients (RR 0.44, 95% CI 0.28–0.79; *p* < 0.0004) [17], but reductions in sepsis failed to reach a significant level within the ICU patient population (RR 0.56, 95% CI 0.26–1.19; *p =* 0.13) [18]. Reductions in 30-day mortality rates failed to reach significance in all three studies, but it is noticeable that a mortality reduction of 54% in the current study (RR 0.46, 95% CI 0.20–1.08; *p* = 0.07) is closer to a significant effect than within ICU populations (RR 0.90; 95% CI 0.69–1.16; *p* = 0.41) [18]. Improved clinical outcomes for PN with ILEs containing fish oil have also been found in many other meta-analyses conducted by a variety of research groups [54,55,56,57,58,59,60,61,62,63]. 

It should be noted, however, that the current study has several inherent weaknesses based on the moderate-to-low quality of most studies in this field. A major limitation is that all of the included clinical trials were conducted at a single center. Another limitation is a lack of reporting in certain studies, leading to ‘some concerns’ in risk-of-bias assessments in the majority of cases. In particular, for the outcome of sepsis, it would be prudent to interpret the study results with caution owing to a relatively low number of trials and variability in reporting (or lack of reporting) of sepsis definitions. Similarly, the interpretation of reductions in length of stay merits careful consideration; while the overall effect appears reassuringly significant, some degree of variation is to be expected across different patient populations requiring parenteral nutrition in hospital wards, as suggested by the observed heterogeneity in effect estimates.

The present study, encompassing 27 randomized controlled trials, has updated the search and meta-analysis for general ward patients that formed a subset of our previous publication on this topic [17]. This updated search and analysis was needed from a methodological perspective: according to guidance from the Cochrane Collaboration, updates to systematic reviews and meta-analyses are advised at intervals of no more than two years, where practicable [21]. Moreover, as there are potential differences in metabolic needs and nutritional requirements between ICU and general ward patients, it is necessary to conduct this analysis for general ward patients to determine if this subgroup benefitted in a similar fashion to previous analyses [17,18]. Furthermore, the current study extended the previous analysis by separately examining the cost-effectiveness within the population hospitalized in a general ward. Despite higher acquisition costs for FO-PN than standard PN, the use of FO-PN was, overall, a cost-saving strategy in the current study for patients hospitalized in a general ward, as has been found for the ICU population [18] and for the overall group of all hospitalized patients (ICU and general ward populations) [64]. It is noticeable that in countries where ward costs are highest, such as the UK, the greatest savings are likely with this strategy.

## 5. Conclusions

In summary, this meta-analysis and cost-effectiveness study confirms and extends previous results. It provides evidence that FO-PN provides significant clinical, non-clinical, and cost-effectiveness benefits over NF-PN within a patient population hospitalized in a general ward.

## Figures and Tables

**Table 1:** Characteristics of the randomized controlled trials included (*n* = 29) [25,26,27,28,29,30,31,32,33,34,35,36,37,38,39,40,41,42,43,44,45,46,47,48,49,50,51,52,53], showing extracted outcomes.

Study	Country	Patient Type (n ^a^)	PN Type	Intervention ^b^	Comparator	Clinical Outcomes ^c^	Laboratory Outcomes
Aliyazicioglu et al., 2013 [25]	UK	Colorectal cancer surgery (n = 36)	total	Standard TPN/FO (FO: 0.10–0.20 g/kg/day; % DLD not available)	Standard TPN	HLOS	–
Al Leswas et al., 2020 [26]	Turkey	Severe acute pancreatitis(n = 45)	total	SO/MCT/FO (FO: 0.20 g/kg/day; 10% DLD)	SO/MCT	Mortality, infections, HLOS, and sepsis	CRP, DHA, EPA, and TNF
Badia-Tahull et al., 2010 [27]	Spain	Major intestinal surgery (n = 29)	PN	SO/OO/FO (FO: 0.12–0.17 g/kg/day; 16.6% DLD)	SO/OO	Mortality, infections, HLOS, and sepsis	ALT, Cr, CRP, GGT, and PU
Chen et al., 2017 [28]	China	Gastric cancer surgery (n = 120)	PN	SO/MCT/OO/FO (FO: 0.15 g/kg/day; 15% DLD)	SO	Infections and HLOS	ALT, bilirubin, CRP, and IL-6
Demirer et al., 2016 [29]	Turkey	Major abdominal surgery (n = 52)	PN	SO/OO/FO (FO dose not available; 15% DLD)	SO/OO or SO/MCT	–	CRP, IL-6, and TNF
Flores-López et al., 2023 [30]	Mexico	Intestinal failure (n = 20)	total	SO/OO + FO (FO: 0.1–0.2 g/kg/day)	SO/OO	Mortality, infections, HLOS, and sepsis	–
Ge et al., 2024 [31]	China	Major abdominal surgery (n = 268)	total	MCT/LCT + FO (FO: 0.2 g/kg/day)	MCT/LCT	Mortality, infections, HLOS, and sepsis	CRP, IL-6, and TNF
Grimm et al., 2006 [32]	Germany	Major abdominal surgery (n = 33)	total	SO/MCT/OO/FO (FO: 0.23 g/kg/day; 15% DLD)	SO	HLOS	alpha-T, DHA, EPA, LTB4, LTB5, and LTB ratio
Hallay et al., 2010 [33]	Hungary	Gastrointestinal surgery (n = 41)	total	SO/MCT/OO/FO(FO: 0.21 g/kg/day; 15% DLD)	SO/MCT	–	ALT, AST, bilirubin, and GGT
Jiang et al., 2010 [34]	China	Gastrointestinal cancer surgery (n = 206)	PN	SO/FO (FO: 0.2 g/kg/day; 17% DLD)	SO	Infections, HLOS, and sepsis	Cr, IL-6, and TNF
Klek et al., 2005 [35]	Poland	Gastric cancer surgery (n = 105, enrolled; n = 71, included in our analysis)	PN	SO/MCT/FO (FO: 0.10 g/kg/day; % DLD not available)	SO/MCT	Infections and HLOS	ALT, AST, Cr, and PU
Klek et al., 2008 [36]	Poland	Gastrectomy or pancreaticoduodenectomy (n = 205)	PN	SO/MCT/FO (plus glutamine) (FO: 0.10 g/kg/day; % DLD not available)	SO/MCT	Mortality, infections, HLOS, and sepsis	–
Klek et al., 2011 [37]	Poland	Gastrectomy or pancreaticoduodenectomy (n = 167)	PN	SO/MCT/FO (plus glutamine) (FO: 0.10 g/kg/day; % DLD not available)	SO/MCT	Mortality, infections, and sepsis	–
Koller et al., 2003 [38]	Germany	Major abdominal surgery (n = 30)	total	SO/MCT/FO (FO: 0.07–0.14 g/kg/day; 10% DLD)	SO	–	LTB4, LTB5, and LTB ratio
Liang et al., 2008 [39]	China	Radical colorectal cancer resection (n = 41)	total	SO/FO (FO: 0.20 g/kg/day; 17% DLD)	SO	Mortality, infection, and HLOS	GGT, IL-6, Plt, and TNF
Linseisen et al., 2000 [40]	Germany	Major abdominal surgery (n = 33)	total	SO/MCT/FO(FO: 0.14 g/kg/day; 10% DLD)	SO	–	alpha-T, DHA, and EPA
Ma et al., 2012 [41]	Taiwan	Gastrointestinal tumor surgery (n = 40)	PN	SO/MCT/OO/FO (FO: 0.15–0.30 g/kg/day; 15% DLD)	SO/MCT	HLOS	ALT, AST, bilirubin, Cr, CRP, IL-6, PU, TG, and TNF
Ma et al., 2015 [42]	Taiwan	Gastric and colorectal cancer surgery (n = 99)	PN	SO/MCT/FO (FO: 0.08–0.15 g/kg/day; 10% DLD)	SO/MCT	Infections	ALT, AST, bilirubin, CRP, GGT, IL-6, TG, and TNF
Makay et al., 2011 [43]	Turkey	Major gastric cancer surgery (n = 26)	PN	SO/FO (FO: 0.2 g/kg/day; 25% DLD)	SO	Mortality, infections, and HLOS	ALT, AST, Cr, and PU
Mertes et al., 2006 [44]	European—Multicenter	Abdominal or thoracic surgery (n = 249)	total	SO/MCT/OO/FO (FO: 0.23 g/kg/day; 15% DLD)	SO	Mortality and HLOS	ALT, AST, bilirubin, GGT, and TG
Schauder et al., 2002 [45]	Germany	Large bowel surgery (n = 60)	total	SO/FO (FO: 0.2 g/kg/day; 17% DLD	SO	–	TNF
Senkal et al., 2007 [46]	Germany	Colorectal surgery (n = 40, received study treatments)	total	SO/MCT/FO (FO: 0.14–0.28 g/kg/day; 10% DLD)	SO/MCT	Infections	DHA, EPA
Wang et al., 2012 [47]	China	Gastrointestinal surgery (n = 64)	total	SO/MCT/FO (FO: 0.04–0.08 g/kg/day; 10% DLD)	SO/MCT	Infections and sepsis	ALT, AST, bilirubin, CRP, GGT, IL-6, LTB ratio, Plt, TG, and TNF
Wei et al., 2014 [48]	China	Surgical resection of gastric tumors (n = 52)	total	SO/FO (FO dose not available; 20% DLD)	SO	Infections	CRP, IL-6, and TNF
Wu et al., 2014 [49]	Taiwan	Gastrointestinal surgery (n = 40)	PN	SO/MCT/OO/FO (FO 0.13 g/kg/day; 15% DLD)	SO/MCT	Infections and HLOS	ALT, AST, bilirubin, Cr, CRP, GGT, IL-6, PU, TG, and TNF
Zhang et al., 2017 [50]	China	Hepatectomy (n = 320)	PN	SO/MCT/FO (FO dose not available; % DLD not available)	SO/MCT	Mortality, infections, HLOS, and sepsis	ALT, bilirubin, Cr, CRP, TG, Plt, and PU
Zhixue et al., 2018 [51]	China	Liver cancer surgery (n = 75)	PN	SO/MCT/FO (FO dose not available; % DLD not available)	SO/MCT	–	IL-6 and TNF
Zhu et al., 2012 [52]	China	Colorectal cancer surgery (n = 57, completed trial)	total	SO/FO (FO: 0.2 g/kg/day; 17% DLD)	SO	Infection, HLOS, and sepsis	IL-6 and TNF
Zhu et al., 2013 [53]	China	Pancreaticoduodenectomy (n = 76)	total	SO/MCT/FO (FO: 0.2 g/kg/day; 18% DLD)	SO/MCT	Mortality, infection, HLOS, and hospital readmission	ALT, AST, and bilirubin

^a^ The number of randomized patients is listed if available, but if was not available, an alternative descriptor was used for the patient population/number. ^b^ This column also shows, in parenthesis, the daily dose of fish oil and the approximate percentage of the daily lipid dose (DLD) supplied by fish oil if these data are available. ^c^ Sepsis outcomes encompassed events classified by the original study’s authors as either septic or consistent with systemic inflammatory response syndrome (SIRS). Alpha-T, alpha-tocopherol; ALT, alanine aminotransferase; AST, aspartate aminotransferase; Cr, serum creatinine; CRP, C-reactive protein; DHA, (%) docosahexaenoic acid content in serum/cellular membranes; EPA, (%) eicosapentaenoic acid content in serum/cellular membranes; FO, fish oil emulsion; GGT, γ-glutamyl transferase; HLOS, hospital length of stay; ICU, intensive care unit; LCT, long-chain triglycerides; LTB, leukotriene B; LTB ratio, LTB5:LTB4; MCT, medium-chain triglycerides; OO, olive oil emulsion; PU, plasma urea; Plt, platelet; SO, soybean oil emulsion; TGs, triglycerides; and TNF, tumor necrosis factor.

**Table 2:** Mean costs (Euro) based on model simulations for parenteral nutrition (PN) containing fish oil and NF-PN: results for France, Germany, Italy, Spain, and the UK.

Country	Cost Type	PN Containing Fish Oil (€)	Standard PN (€)	Difference (€)	ICER
France	Ward cost	10,054	11,617	−1563	
	Infection cost	166	264	−98	
	Treatment cost	179	181	−2	
	Total	10,399	12,061	−1662	dominant
Germany	Ward cost	7444	8601	−1157	
	Infection cost	286	455	−169	
	Treatment cost	580	687	−107	
	Total	8310	9743	−1433	dominant
Italy	Ward cost	8369	9670	−1301	
	Infection cost	265	421	−156	
	Treatment cost	409	693	−284	
	Total	9043	10,784	−1741	dominant
Spain	Ward cost	7807	9020	−1213	
	Infection cost	298	473	−175	
	Treatment cost	88	150	−62	
	Total	8192	9643	−1451	dominant
UK	Ward cost	12,309	14,223	−1914	
	Infection cost	125	198	−73	
	Treatment cost	327	386	−59	
	Total	12,761	14,807	−2046	dominant

ICER, incremental cost-effectiveness ratio; FA, fatty acid; and PN, parenteral nutrition.

## Data Availability

All data generated or analyzed during this study are included in this published article (and its Supplementary Information Files).

## Supplementary Materials

The following supporting information can be downloaded at: https://www.mdpi.com/article/10.3390/nu17071284/s1, Figure S1. Risk of bias assessment results (A1. Individual study results, traffic light plot; A2. Infection rate: weighed summary, B2. Mortality rate: weighed summary, C2. Length of stay: weighed summary, D2. Sepsis: summary results); Figure S2a. Scatterplot of 1000 incremental cost-effectiveness ration (ICER) estimates in a probabilistic sensitivity analysis (PSA) for France, and associated tabulated data; Figure S2b. Deterministic sensitivity analyses (DSA) results (tornado plots) representing the sensitivity of cost savings with omega-3 fatty-acid enriched parenteral nutrition to variation in key parameters: patients in France; Figure S3a. Scatterplot of 1000 incremental cost-effectiveness ration (ICER) estimates in a probabilistic sensitivity analysis (PSA) for Germany, and associated tabulated data; Figure S3b. Deterministic sensitivity analyses (DSA) results (tornado plots) representing the sensitivity of cost savings with omega-3 fatty-acid enriched parenteral nutrition to variation in key parameters: patients in Germany; Figure S4a. Scatterplot of 1000 incremental cost-effectiveness ration (ICER) estimates in a probabilistic sensitivity analysis (PSA) for Italy, and associated tabulated data; Figure S4b. Deterministic sensitivity analyses (DSA) results (tornado plots) representing the sensitivity of cost savings with omega-3 fatty-acid enriched parenteral nutrition to variation in key parameters: patients in Italy; Figure S5a. Scatterplot of 1000 incremental cost-effectiveness ration (ICER) estimates in a probabilistic sensitivity analysis (PSA) for Spain, and associated tabulated data; Figure S5b. Deterministic sensitivity analyses (DSA) results (tornado plots) representing the sensitivity of cost savings with omega-3 fatty-acid enriched parenteral nutrition to variation in key parameters: patients in Spain; Figure S6a. Scatterplot of 1000 incremental cost-effectiveness ration (ICER) estimates in a probabilistic sensitivity analysis (PSA) for the UK, and associated tabulated data; Figure S6b. Deterministic sensitivity analyses (DSA) results (tornado plots) representing the sensitivity of cost savings with omega-3 fatty-acid enriched parenteral nutrition to variation in key parameters: patients in the UK; Table S1. Search strings used for MEDLINE (PubMed interface), EMBASE (Elsevier interface), and Web of Science Core Collection (WOS); Table S2. Summary of meta-analysis results for laboratory parameters; Table S3. Comparison of clinical outcomes and cost-effectiveness results from previous meta-analyses and the current study.

## References

[1] Saunders J., Smith T. (2010). Malnutrition: Causes and Consequences. Clin. Med..

[2] Correia M.I.T.D., Perman M.I., Waitzberg D.L. (2017). Hospital Malnutrition in Latin America: A Systematic Review. Clin. Nutr..

[3] Singer P., Blaser A.R., Berger M.M., Alhazzani W., Calder P.C., Casaer M.P., Hiesmayr M., Mayer K., Montejo J.C., Pichard C. (2019). ESPEN Guideline on Clinical Nutrition in the Intensive Care Unit. Clin. Nutr. Edinb. Scotl..

[4] Mayer K., Schaefer M.B., Hecker M. (2019). Intravenous N-3 Fatty Acids in the Critically Ill. Curr. Opin. Clin. Nutr. Metab. Care.

[5] Mayer K., Klek S., García-de-Lorenzo A., Rosenthal M.D., Li A., Evans D.C., Muscaritoli M., Martindale R.G. (2020). Lipid Use in Hospitalized Adults Requiring Parenteral Nutrition. J. Parenter. Enter. Nutr..

[6] Raman M., Almutairdi A., Mulesa L., Alberda C., Beattie C., Gramlich L. (2017). Parenteral Nutrition and Lipids. Nutrients.

[7] Calder P.C., Adolph M., Deutz N.E., Grau T., Innes J.K., Klek S., Lev S., Mayer K., Michael-Titus A.T., Pradelli L. (2018). Lipids in the Intensive Care Unit: Recommendations from the ESPEN Expert Group. Clin. Nutr..

[8] Waitzberg D.L., Torrinhas R.S., Jacintho T.M. (2006). New Parenteral Lipid Emulsions for Clinical Use. J. Parenter. Enter. Nutr..

[9] Calder P.C. (2006). Use of Fish Oil in Parenteral Nutrition: Rationale and Reality. Proc. Nutr. Soc..

[10] Calder P.C., Waitzberg D.L., Klek S., Martindale R.G. (2020). Lipids in Parenteral Nutrition: Biological Aspects. J. Parenter. Enter. Nutr..

[11] Martindale R.G., Berlana D., Boullata J.I., Cai W., Calder P.C., Deshpande G.H., Evans D., Garcia-de-Lorenzo A., Goulet O.J., Li A. (2020). Summary of Proceedings and Expert Consensus Statements From the International Summit “Lipids in Parenteral Nutrition”. J. Parenter. Enter. Nutr..

[12] Serhan C.N. (2014). Pro-Resolving Lipid Mediators Are Leads for Resolution Physiology. Nature.

[13] Bird J.K., Troesch B., Warnke I., Calder P.C. (2021). The Effect of Long Chain Omega-3 Polyunsaturated Fatty Acids on Muscle Mass and Function in Sarcopenia: A Scoping Systematic Review and Meta-Analysis. Clin. Nutr. ESPEN.

[14] McGlory C., Calder P.C., Nunes E.A. (2019). The Influence of Omega-3 Fatty Acids on Skeletal Muscle Protein Turnover in Health, Disuse, and Disease. Front. Nutr..

[15] Williams J., Tu S., Lodhia C., Gu G., Haar G., O’Connor J., Niewiadomski O., Tandiari T., Nicoll A.J. (2019). Parenteral Nutrition: How Do Patients Initiated in the Intensive Care Unit Differ from Those on the Ward?. Clin. Nutr. Exp..

[16] Weimann A., Braga M., Carli F., Higashiguchi T., Hübner M., Klek S., Laviano A., Ljungqvist O., Lobo D.N., Martindale R.G. (2021). ESPEN Practical Guideline: Clinical Nutrition in Surgery. Clin. Nutr..

[17] Pradelli L., Mayer K., Klek S., Omar Alsaleh A.J., Clark R.A.C., Rosenthal M.D., Heller A.R., Muscaritoli M. (2020). Ω-3 Fatty-Acid Enriched Parenteral Nutrition in Hospitalized Patients: Systematic Review With Meta-Analysis and Trial Sequential Analysis. J. Parenter. Enter. Nutr..

[18] Pradelli L., Klek S., Mayer K., Omar Alsaleh A.J., Rosenthal M.D., Heller A.R., Muscaritoli M. (2020). Omega-3 Fatty Acid-Containing Parenteral Nutrition in ICU Patients: Systematic Review with Meta-Analysis and Cost-Effectiveness Analysis. Crit. Care.

[19] PROSPERO International Register of Prospective Systematic Reviews. https://www.crd.york.ac.uk/prospero/display_record.php?ID=CRD42021293972.

[20] Liberati A., Altman D.G., Tetzlaff J., Mulrow C., Gotzsche P.C., Ioannidis J.P.A., Clarke M., Devereaux P.J., Kleijnen J., Moher D. (2009). The PRISMA Statement for Reporting Systematic Reviews and Meta-Analyses of Studies That Evaluate Healthcare Interventions: Explanation and Elaboration. BMJ.

[21] Higgins J.P., Green S. (2008). Cochrane Handbook for Systematic Reviews of Interventions: Cochrane Book Series.

[22] Moher D., Shamseer L., Clarke M., Ghersi D., Liberati A., Petticrew M., Shekelle P., Stewart L.A., PRISMA-P Group (2015). Preferred Reporting Items for Systematic Review and Meta-Analysis Protocols (PRISMA-P) 2015 Statement. Syst. Rev..

[23] Mitchell M., Muftakhidinov B., Winchen T., Wilms A., van Schaik B., Badger T.G., Jędrzejewski-Szmek Z. Engauge Digitizer Software 2020. https://sourceforge.net/projects/digitizer/.

[24] Wan X., Wang W., Liu J., Tong T. (2014). Estimating the Sample Mean and Standard Deviation from the Sample Size, Median, Range and/or Interquartile Range. BMC Med. Res. Methodol..

[25] Aliyazicioglu T., Cantürk N.Z., Şimşek T., Kolayli F., Çekmen M. (2013). Effects of Standard and/or Glutamine Dipeptide and/or Omega-3 Fatty Acid-Supplemented Parenteral Nutrition on Neutrophil Functions, Interleukin-8 Level, and Length of Stay: A Double-Blind, Controlled, Randomized Study. East Afr. Med. J..

[26] Al-Leswas D., Eltweri A.M., Chung W.-Y., Arshad A., Stephenson J.A., Al-Taan O., Pollard C., Fisk H.L., Calder P.C., Garcea G. (2020). Intravenous Omega-3 Fatty Acids Are Associated with Better Clinical Outcome and Less Inflammation in Patients with Predicted Severe Acute Pancreatitis: A Randomised Double Blind Controlled Trial. Clin. Nutr..

[27] Badía-Tahull M.B., Llop-Talaverón J.M., Leiva-Badosa E., Biondo S., Farran-Teixidó L., Ramón-Torrell J.M., Jódar-Masanes R. (2010). A Randomised Study on the Clinical Progress of High-Risk Elective Major Gastrointestinal Surgery Patients Treated with Olive Oil-Based Parenteral Nutrition with or without a Fish Oil Supplement. Br. J. Nutr..

[28] Chen H., Pan D., Li L. (2017). The Effects of Multi-Oil Fat Emulsion on Older Patients with Gastric Cancer. Biomed Res.

[29] Demirer S., Sapmaz A., Karaca A.S., Kepenekci I., Aydintug S., Balci D., Sonyurek P., Kose K. (2016). Effects of Postoperative Parenteral Nutrition with Different Lipid Emulsions in Patients Undergoing Major Abdominal Surgery. Ann. Surg. Treat. Res..

[30] Flores-López A., Guevara-Cruz M., Avila-Nava A., González-Garay A.G., González-Salazar L.E., Reyes-Ramírez A.L., Pedraza-Chaverri J., Medina-Campos O.N., Medina-Vera I., Reyes-García J.G. (2023). N-3 Polyunsaturated Fatty Acid Supplementation Affects Oxidative Stress Marker Levels in Patients with Type II Intestinal Failure: A Randomized Double Blind Trial. Antioxidants.

[31] Ge X., Liu H., Wu Y., Liu W., Qi W., Ye L., Cao Q., Lian H., Bai R., Zhou W. (2024). Parenteral n–3 Polyunsaturated Fatty Acids Supplementation Improves Postoperative Recovery for Patients with Crohn’s Disease after Bowel Resection: A Randomized, Unblinded Controlled Clinical Trial. Am. J. Clin. Nutr..

[32] Grimm H., Mertes N., Goeters C., Schlotzer E., Mayer K., Grimminger F., Fürst P. (2006). Improved Fatty Acid and Leukotriene Pattern with a Novel Lipid Emulsion in Surgical Patients. Eur. J. Nutr..

[33] Hallay J., Olah A.V., Fulesdi B., Kocsor M., Vegh T., Kovacs G., Takacs I., Sapy P., Nagy D., Telessy I.G. (2010). Hepatobiliary Response in Postoperative Lipid Therapy in Gastrointestinal Surgery. Hepatogastroenterology.

[34] Jiang Z.M., Wilmore D.W., Wang X.R., Wei J.M., Zhang Z.T., Gu Z.Y., Wang S., Han S.M., Jiang H., Yu K. (2010). Randomized Clinical Trial of Intravenous Soybean Oil Alone versus Soybean Oil plus Fish Oil Emulsion after Gastrointestinal Cancer Surgery. Br. J. Surg..

[35] Klek S., Kulig J., Szczepanik A.M., Jedrys J., Kotodziejczyk P. (2005). The Clinical Value of Parenteral Immunonutrition in Surgical Patients. Acta Chir. Belg..

[36] Klek S., Kulig J., Sierzega M., Szybinski P., Szczepanek K., Kubisz A., Kowalczyk T., Gach T., Pach R., Szczepanik A.M. (2008). The Impact of Immunostimulating Nutrition on Infectious Complications After Upper Gastrointestinal Surgery: A Prospective, Randomized, Clinical Trial. Ann. Surg..

[37] Klek S., Sierzega M., Szybinski P., Szczepanek K., Scislo L., Walewska E., Kulig J. (2011). Perioperative Nutrition in Malnourished Surgical Cancer Patients–A Prospective, Randomized, Controlled Clinical Trial. Clin. Nutr..

[38] Köller M. (2003). Impact of Omega-3 Fatty Acid Enriched TPN on Leukotriene Synthesis by Leukocytes after Major Surgery. Clin. Nutr..

[39] Liang B., Wang S., Ye Y.-J., Yang X.-D., Wang Y.-L., Qu J., Xie Q.-W., Yin M.-J. (2008). Impact of Postoperative Omega-3 Fatty Acid-Supplemented Parenteral Nutrition on Clinical Outcomes and Immunomodulations in Colorectal Cancer Patients. World J. Gastroenterol..

[40] Linseisen J., Hoffmann J., Lienhard S., Jauch K.-W., Wolfram G. (2000). Antioxidant Status of Surgical Patients Receiving TPN with an Ω-3-Fatty Acid-Containing Lipid Emulsion Supplemented with α-Tocopherol. Clin. Nutr..

[41] Ma C., Sun L., Chen F., Lu C., Shih Y., Tsai H., Chuang J., Wang J. (2012). A Double-Blind Randomized Study Comparing the Efficacy and Safety of a Composite vs a Conventional Intravenous Fat Emulsion in Postsurgical Gastrointestinal Tumor Patients. Nutr. Clin. Pract..

[42] Ma C.-J., Wu J.-M., Tsai H.-L., Huang C.-W., Lu C.-Y., Sun L.-C., Shih Y.-L., Chen C.-W., Chuang J.-F., Wu M.-H. (2015). Prospective Double-Blind Randomized Study on the Efficacy and Safety of an n-3 Fatty Acid Enriched Intravenous Fat Emulsion in Postsurgical Gastric and Colorectal Cancer Patients. Nutr. J..

[43] Makay O., Kaya T., Firat O., Sozbilen M., Caliskan C., Gezer G., Uyar M., Ersin S. (2011). Ω-3 Fatty Acids Have No Impact on Serum Lactate Levels After Major Gastric Cancer Surgery. J. Parenter. Enter. Nutr..

[44] Mertes N., Grimm H., Fürst P., Stehle P. (2006). Safety and Efficacy of a New Parenteral Lipid Emulsion (SMOFlipid) in Surgical Patients: A Randomized, Double-Blind, Multicenter Study. Ann. Nutr. Metab..

[45] Schauder P., Röhn U., Schäfer G., Korff G., Schenk H.-D. (2002). Impact of Fish Oil Enriched Total Parenteral Nutrition on DNA Synthesis, Cytokine Release and Receptor Expression by Lymphocytes in the Postoperative Period. Br. J. Nutr..

[46] Senkal M., Geier B., Hannemann M., Deska T., Linseisen J., Wolfram G., Adolph M. (2007). Supplementation of Ω-3 Fatty Acids in Parenteral Nutrition Beneficially Alters Phospholipid Fatty Acid Pattern. J. Parenter. Enter. Nutr..

[47] Wang J., Yu J.-C., Kang W.-M., Ma Z.-Q. (2012). Superiority of a Fish Oil–Enriched Emulsion to Medium-Chain Triacylglycerols/Long-Chain Triacylglycerols in Gastrointestinal Surgery Patients: A Randomized Clinical Trial. Nutrition.

[48] Wei Z., Wang W., Chen J., Yang D., Yan R., Cai Q. (2014). A Prospective, Randomized, Controlled Study of ω-3 Fish Oil Fat Emulsion-Based Parenteral Nutrition for Patients Following Surgical Resection of Gastric Tumors. Nutr. J..

[49] Wu M., Wang M., Yang C., Kuo M., Lin M. (2014). Randomized Clinical Trial of New Intravenous Lipid (SMOFlipid 20%) Versus Medium-Chain Triglycerides/Long-Chain Triglycerides in Adult Patients Undergoing Gastrointestinal Surgery. J. Parenter. Enter. Nutr..

[50] Zhang B., Wei G., Li R., Wang Y., Yu J., Wang R., Xiao H., Wu C., Leng C., Zhang B. (2017). N-3 Fatty Acid-Based Parenteral Nutrition Improves Postoperative Recovery for Cirrhotic Patients with Liver Cancer: A Randomized Controlled Clinical Trial. Clin. Nutr..

[51] Zhixue G., Changqing G., Bing H., Zengwang Q., Yanjie K., Ya Z., Weihong W., Liqing T., Xin F., Xiaoyong M. (2018). Effects of Parenteral Nutrition of ω-3 Polyunsaturated Fatty Acid, Arginine and Glutamine on Cellular Immune Status of Patients Following Liver Cancer Surgery. Trop. J. Pharm. Res..

[52] Zhu M.-W., Tang D.-N., Hou J., Wei J.-M., Hua B., Sun J.-H., Cui H.-Y. (2012). Impact of Fish Oil Enriched Total Parenteral Nutrition on Elderly Patients after Colorectal Cancer Surgery. Chin. Med. J..

[53] Zhu X., Wu Y., Qiu Y., Jiang C., Ding Y. (2013). Effect of Parenteral Fish Oil Lipid Emulsion in Parenteral Nutrition Supplementation Combined With Enteral Nutrition Support in Patients Undergoing Pancreaticoduodenectomy. J. Parenter. Enter. Nutr..

[54] Notz Q., Lee Z.-Y., Menger J., Elke G., Hill A., Kranke P., Roeder D., Lotz C., Meybohm P., Heyland D.K. (2022). Omega-6 Sparing Effects of Parenteral Lipid Emulsions—An Updated Systematic Review and Meta-Analysis on Clinical Outcomes in Critically Ill Patients. Crit. Care.

[55] Wei C., Hua J., Bin C., Klassen K. (2010). Impact of Lipid Emulsion Containing Fish Oil on Outcomes of Surgical Patients: Systematic Review of Randomized Controlled Trials from Europe and Asia. Nutrition.

[56] Chen B., Zhou Y., Yang P., Wan H., Wu X. (2010). Safety and Efficacy of Fish Oil–Enriched Parenteral Nutrition Regimen on Postoperative Patients Undergoing Major Abdominal Surgery: A Meta-Analysis of Randomized Controlled Trials. J. Parenter. Enter. Nutr..

[57] Pradelli L., Mayer K., Muscaritoli M., Heller A.R. (2012). Correction: N-3 Fatty Acid-Enriched Parenteral Nutrition Regimens in Elective Surgical and ICU Patients: A Meta-Analysis. Crit. Care.

[58] Palmer A.J., Ho C.K.M., Ajibola O., Avenell A. (2013). The Role of ω-3 Fatty Acid Supplemented Parenteral Nutrition in Critical Illness in Adults: A Systematic Review and Meta-Analysis. Crit. Care Med..

[59] Li N.-N., Zhou Y., Qin X.-P., Chen Y., He D., Feng J.-Y., Wu X.-T. (2014). Does Intravenous Fish Oil Benefit Patients Post-Surgery? A Meta-Analysis of Randomised Controlled Trials. Clin. Nutr..

[60] Manzanares W., Langlois P.L., Dhaliwal R., Lemieux M., Heyland D.K. (2015). Intravenous Fish Oil Lipid Emulsions in Critically Ill Patients: An Updated Systematic Review and Meta-Analysis. Crit. Care.

[61] Bae H.J., Lee G.Y., Seong J.-M., Gwak H.S. (2017). Outcomes with Perioperative Fat Emulsions Containing Omega-3 Fatty Acid: A Meta-Analysis of Randomized Controlled Trials. Am. J. Health. Syst. Pharm..

[62] Kreymann K.G., Heyland D.K., De Heer G., Elke G. (2018). Intravenous Fish Oil in Critically Ill and Surgical Patients–Historical Remarks and Critical Appraisal. Clin. Nutr..

[63] Xu X.-T., Huang H., Tian M.-X., Hu R.-C., Dai Z., Jin X. (2021). A Four-Oil Intravenous Lipid Emulsion Improves Markers of Liver Function, Triglyceride Levels and Shortens Length of Hospital Stay in Adults: A Systematic Review and Meta-Analysis. Nutr. Res..

[64] Pradelli L., Muscaritoli M., Klek S., Martindale R.G. (2020). Pharmacoeconomics of Parenteral Nutrition with Ω-3 Fatty Acids in Hospitalized Adults. J. Parenter. Enter. Nutr..

