# Supplementary material for: Parenteral Nutrition Containing Fish Oil for Hospitalized Non-Intensive Care Unit (ICU) Patients: A Systematic Review, Meta-Analysis, and Cost-Effectiveness Analysis
Source: Nutrients. 2025 Apr 7;17(7):1284. doi: 10.3390/nu17071284 (PMC11990895; doi:10.3390/nu17071284)
Supplement: Supplementary file 1 [file nutrients-17-01284-s001.zip › nutrients-3557992-supplementary.pdf]

Figure S1. Risk of bias assessment results

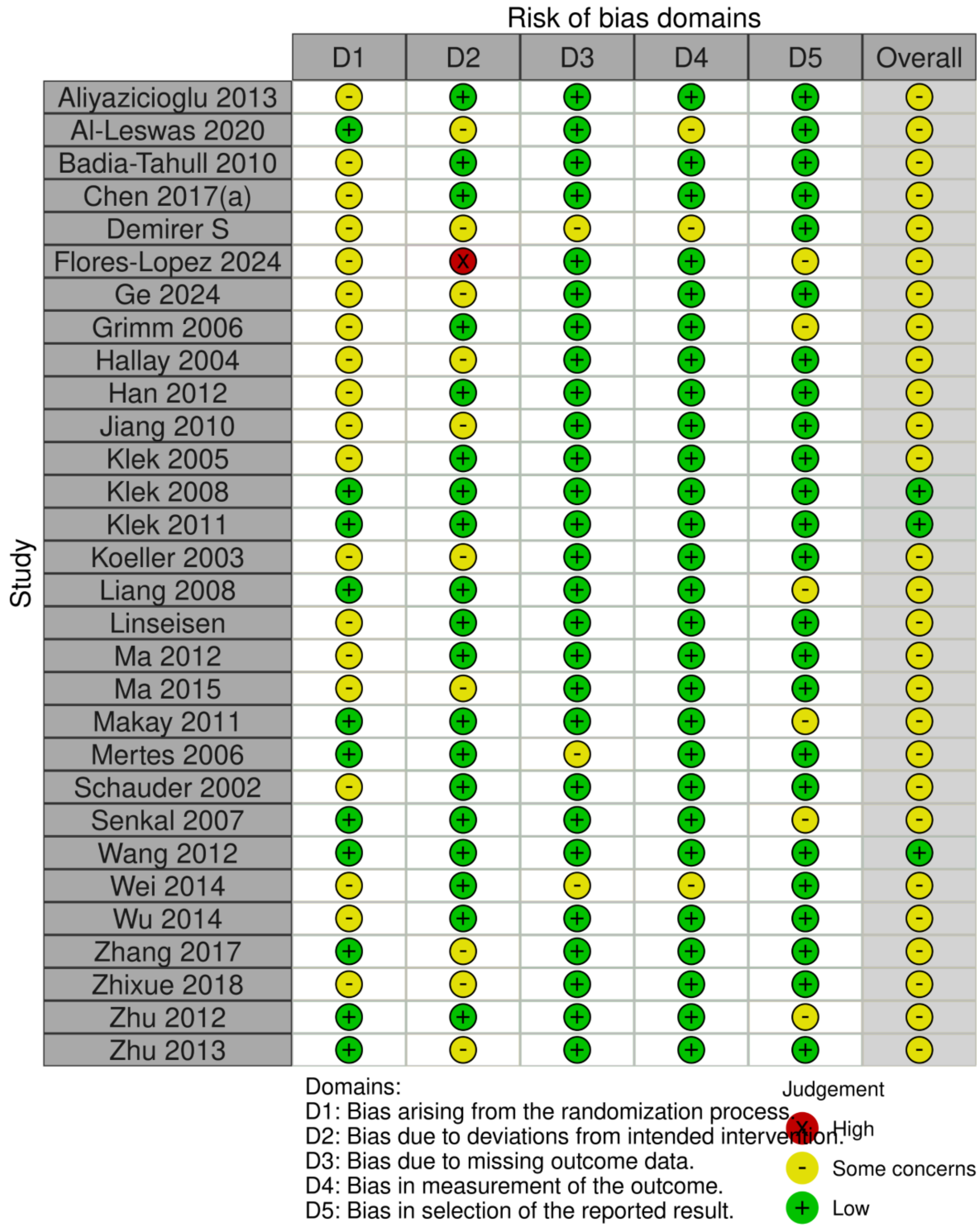

A1. : Individual study results, traffic light plot

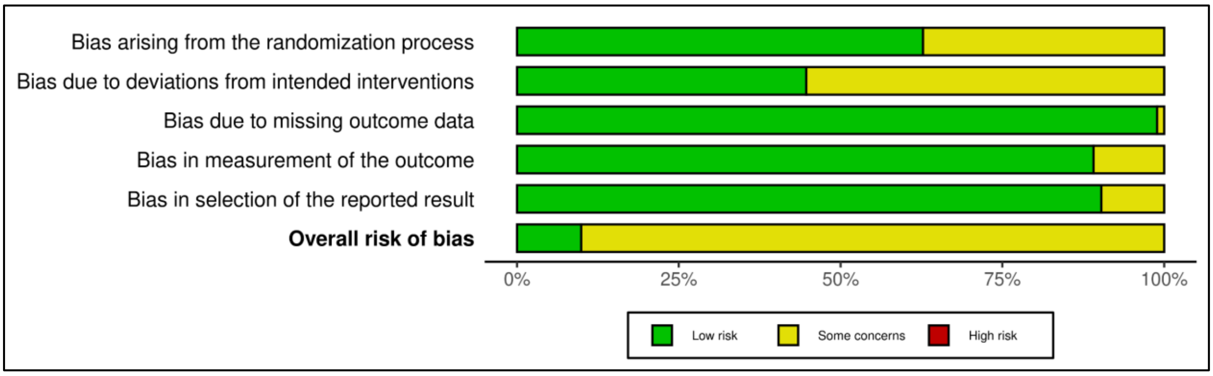

A2. Infection rate: weighed summary.

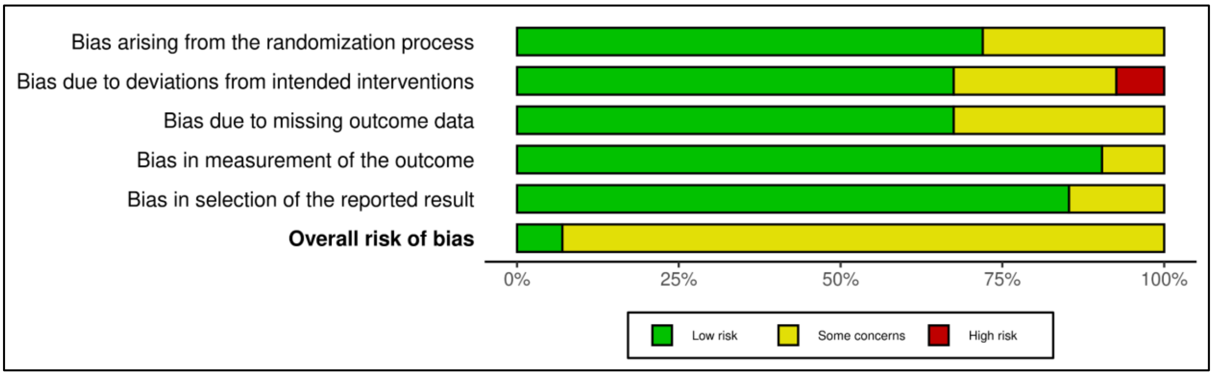

B2. Mortality rate: weighed summary

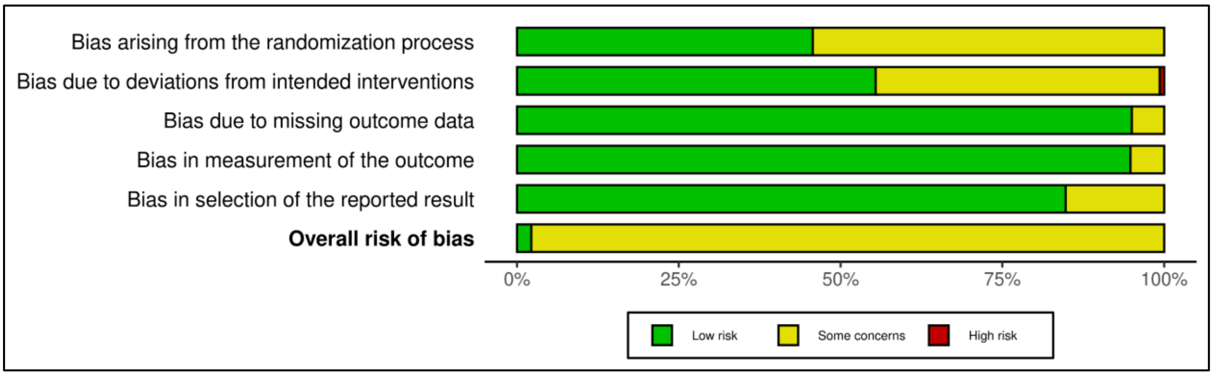

C2. Length of stay: weighed summary

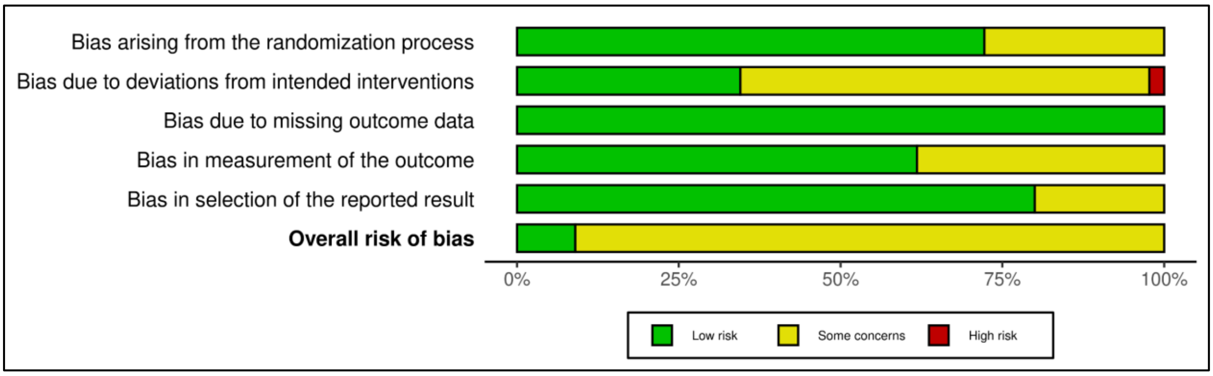

D2. Sepsis: summary results.

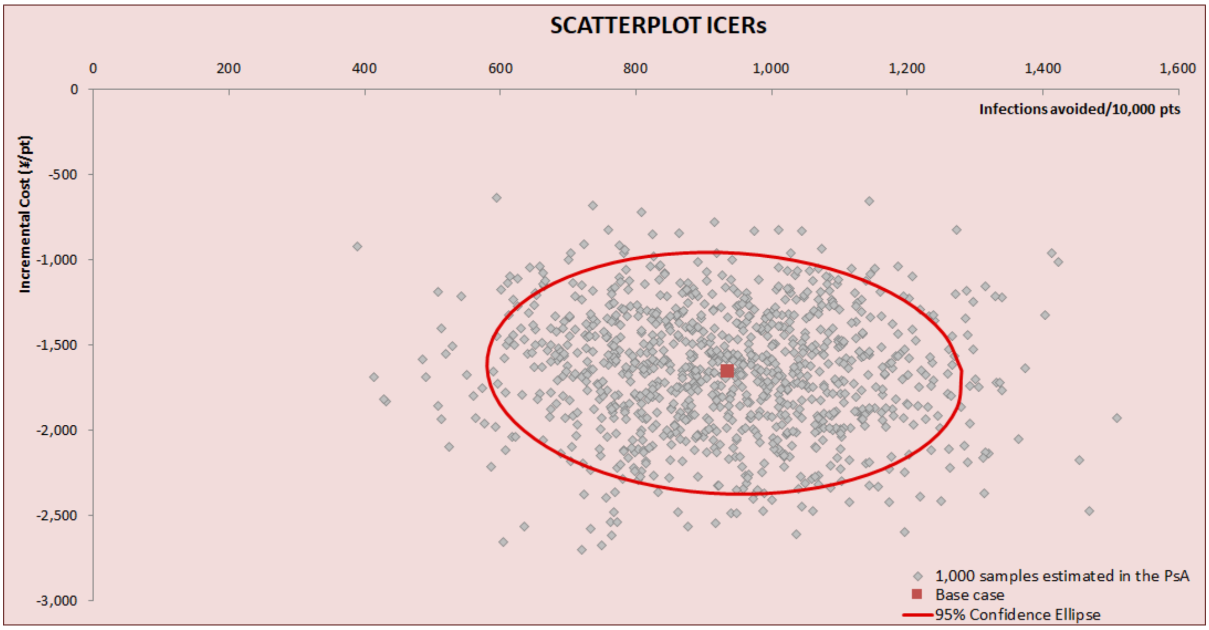

	Mean	95% CI – LL	95% CI – UL
Avoided infections	0.084	0.050	0.121
Incremental cost	-1,671 €	-2,347 €	-1,056 €
**Dominance**			100%

CI – LL, confidence interval lower limit; CI – UL, confidence interval upper limit.

Figure S2a. Scatterplot of 1000 incremental cost-effectiveness ration (ICER) estimates in a probabilistic sensitivity analysis (PSA) for France, and associated tabulated data.

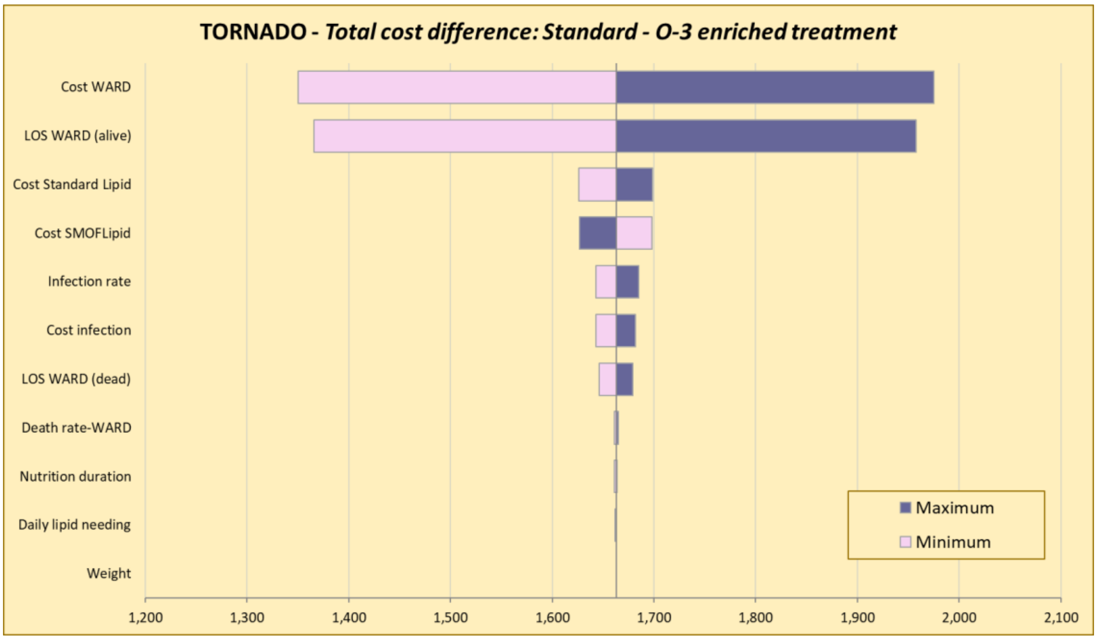

Figure S2b. Deterministic sensitivity analyses (DSA) results (tornado plots) representing the sensitivity of cost savings with omega-3 fatty-acid enriched parenteral nutrition to variation in key parameters: patients in France.

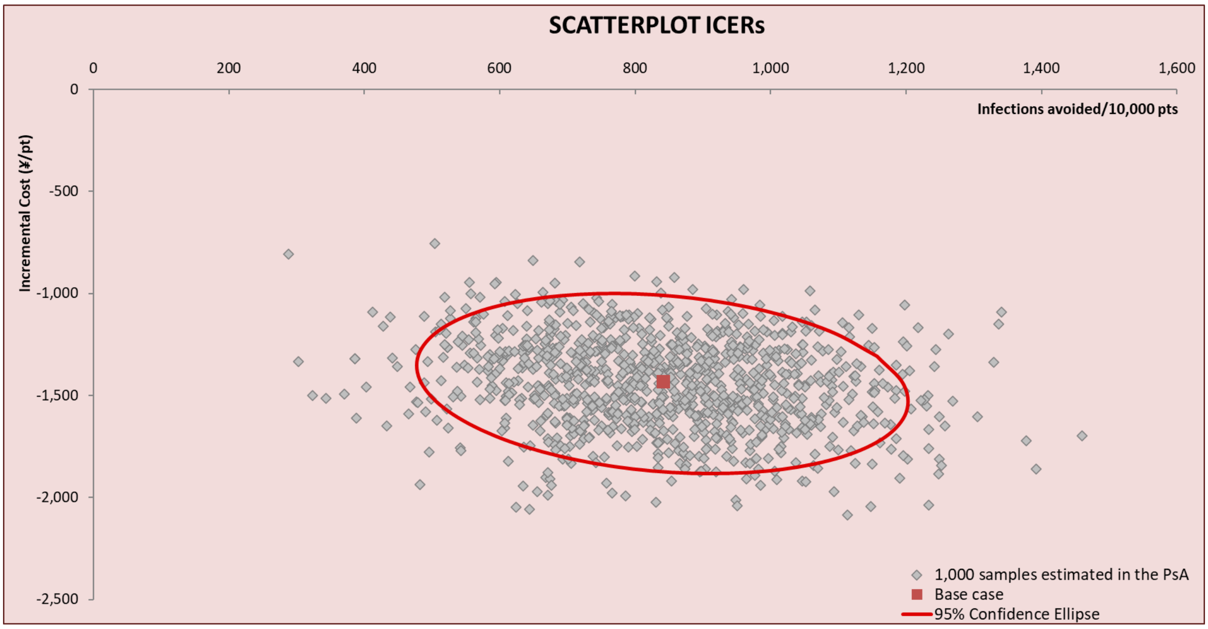

	Mean	95% CI – LL	95% CI – UL
Avoided infections	0.084	0.050	0.121
Incremental cost	-1,441 €	-1,902 €	-1,038 €
**Dominance**			100%

CI – LL, confidence interval lower limit; CI – UL, confidence interval upper limit.

Figure S3a. Scatterplot of 1000 incremental cost-effectiveness ration (ICER) estimates in a probabilistic sensitivity analysis (PSA) for Germany, and associated tabulated data.

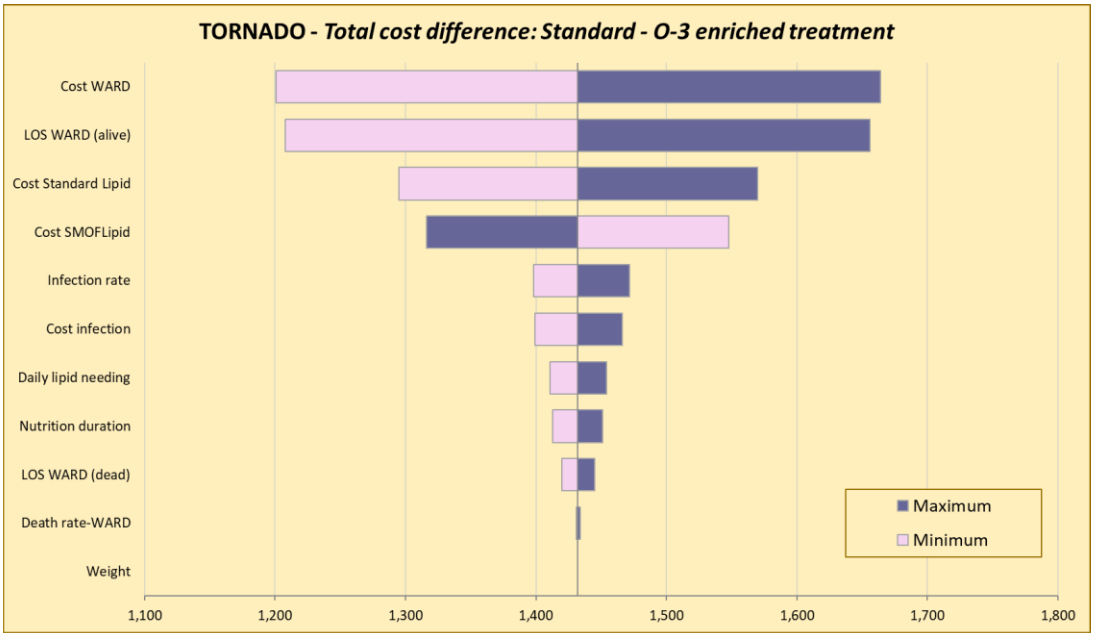

Figure S3b. Deterministic sensitivity analyses (DSA) results (tornado plots) representing the sensitivity of cost savings with omega-3 fatty-acid enriched parenteral nutrition to variation in key parameters: patients in Germany.

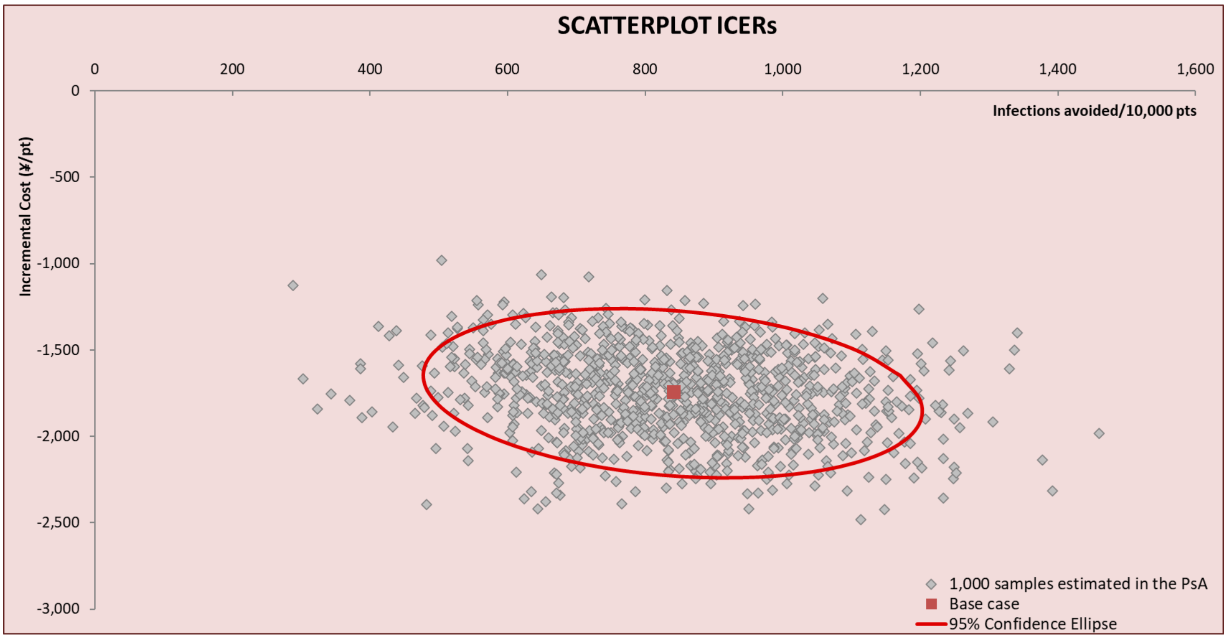

	Mean	95% CI – LL	95% CI – UL
Avoided infections	0.084	0.050	0.121
Incremental cost	-1,751 €	-2,250 €	-1,297 €
**Dominance**			100%

CI – LL, confidence interval lower limit; CI – UL, confidence interval upper limit.

Figure S4a. Scatterplot of 1000 incremental cost-effectiveness ration (ICER) estimates in a probabilistic sensitivity analysis (PSA) for Italy, and associated tabulated data.

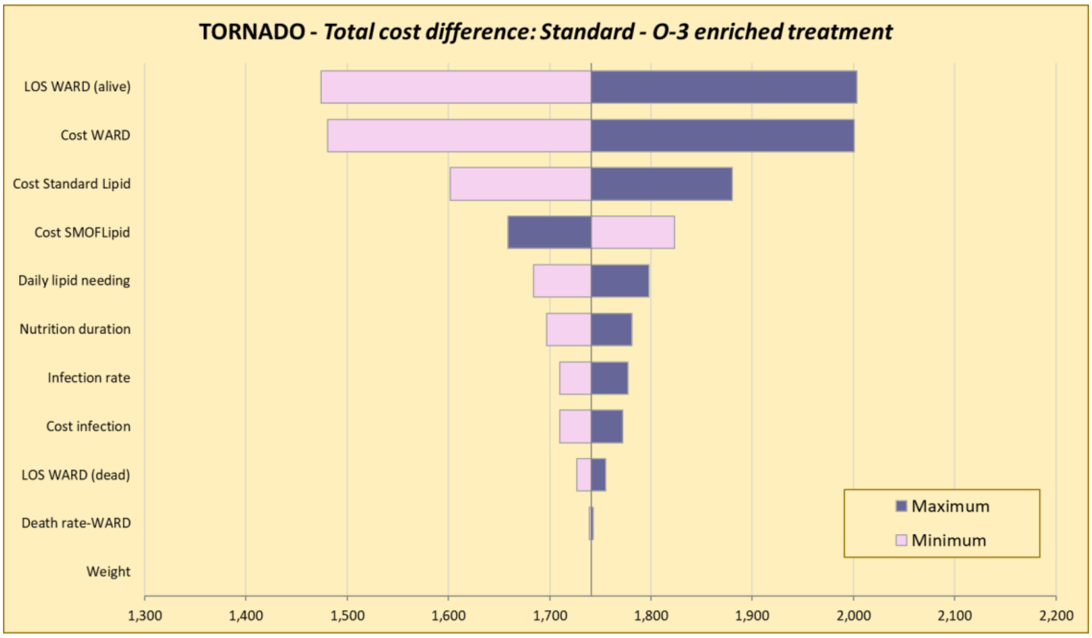

Figure S4b. Deterministic sensitivity analyses (DSA) results (tornado plots) representing the sensitivity of cost savings with omega-3 fatty-acid enriched parenteral nutrition to variation in key parameters: patients in Italy.

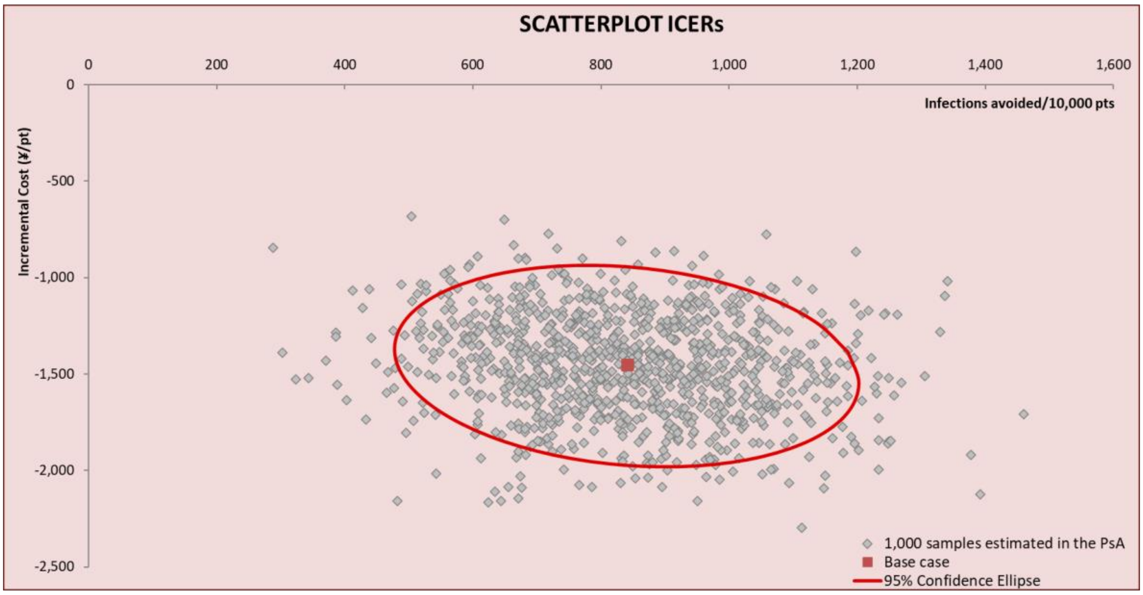

	Mean	95% CI – LL	95% CI –UL
Avoided infections	0.084	0.050	0.121
Incremental cost	-1,458 €	-2,000 €	-975 €
**Dominance**			100%

CI – LL, confidence interval lower limit; CI – UL, confidence interval upper limit.

Figure S5a. Scatterplot of 1000 incremental cost-effectiveness ration (ICER) estimates in a probabilistic sensitivity analysis (PSA) for Spain, and associated tabulated data.

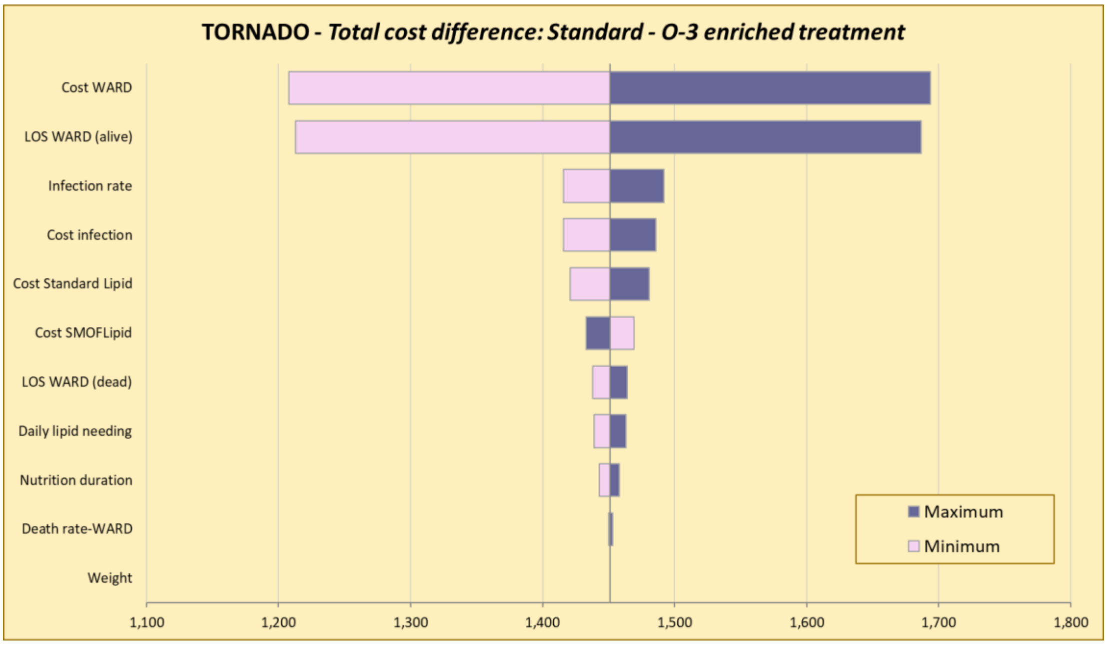

Figure S5b. Deterministic sensitivity analyses (DSA) results (tornado plots) representing the sensitivity of cost savings with omega-3 fatty-acid enriched parenteral nutrition to variation in key parameters: patients in Spain.

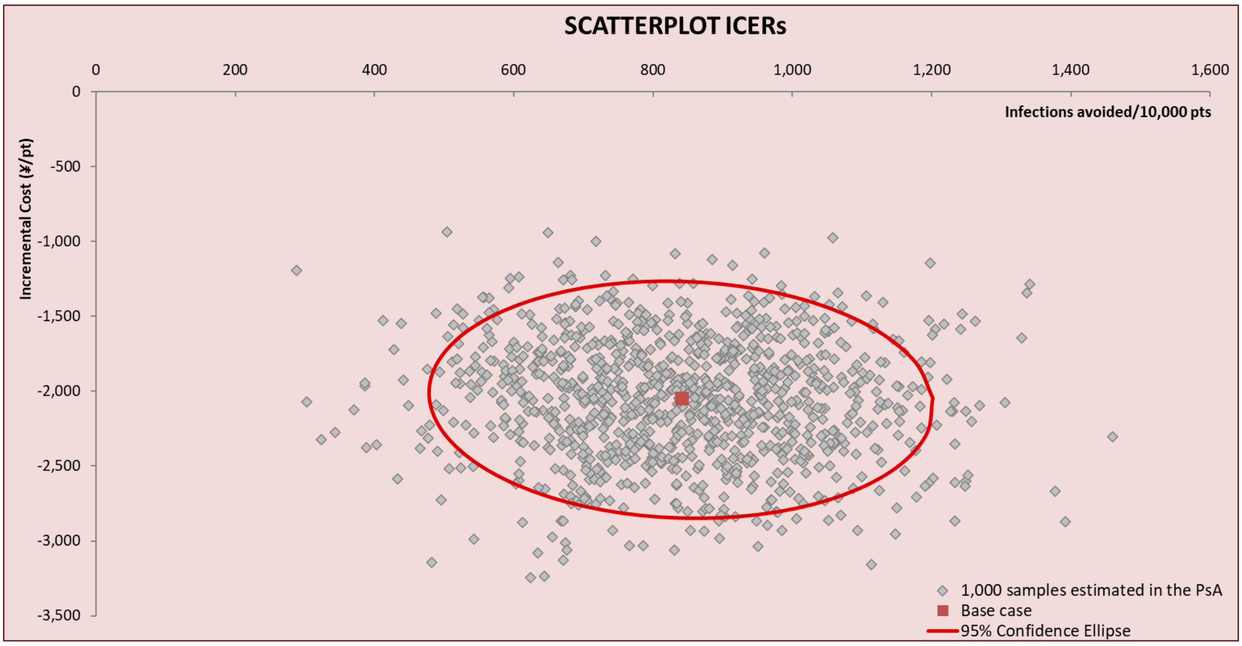

	Mean	95% CI – LL	95% CI – UL
Avoided infections	0.084	0.050	0.121
Incremental cost	-2,056 €	-2,868 €	-1,333 €
**Dominance**			100%

CI – LL, confidence interval lower limit; CI – UL, confidence interval upper limit.

Figure S6a. Scatterplot of 1000 incremental cost-effectiveness ration (ICER) estimates in a probabilistic sensitivity analysis (PSA) for the UK, and associated tabulated data.

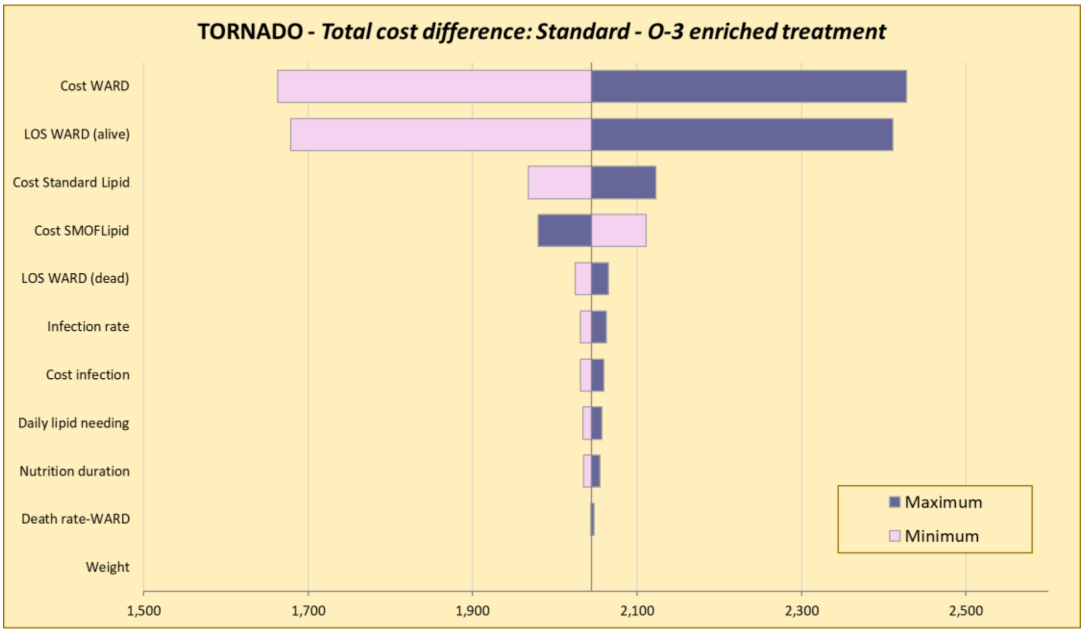

Figure S6b. Deterministic sensitivity analyses (DSA) results (tornado plots) representing the sensitivity of cost savings with omega-3 fatty-acid enriched parenteral nutrition to variation in key parameters: patients in the UK.

Table S1. Search strings used for MEDLINE (PubMed interface), EMBASE (Elsevier interface), and Web of Science Core Collection (WOS).

We searched MEDLINE (PubMed interface), EMBASE (Elsevier interface), and Web of Science Core Collection (WOS) in order to identify randomized trials. The time interval of inclusion was from *any date* until 30 April 2022. The keywords for the search were: *“Parenteral nutrition”, “Fish oil”, “lipids”, “emulsion”, and “randomized controlled trial”.* According to their peculiarities, each database was searched with a specific string developed on these keywords.

MEDLINE

Medline was searched via the PubMed search engine. The following string (no limits[a]) represents a draft MEDLINE search strategy:

1. (parenteral* OR parenteral[tiab] OR "Parenteral Nutrition, Total"[Mesh] OR "Parenteral Nutrition Solutions"[Mesh]) AND
2. (fish* OR fish[tiab] OR oil* OR omeg* OR omeg*[tiab] OR n-3[tiab] OR "n-3" OR "Fish Oils"[Mesh] OR "Fatty Acids, Omega-3"[Mesh] OR
3. lipid[tiab] OR emulsion* OR emulsion[tiab] OR "Fat Emulsions, Intravenous"[Mesh] OR "Lipids"[Mesh] OR “Immunonutrition”[tiab]) AND
4. (Random* OR Random*[tiab] OR "Randomized Controlled Trials as Topic"[Mesh] OR "Randomized Controlled Trial" [Publication Type])

EMBASE

The following string represents a draft EMBASE search strategy (no limits[a]):

1. ((parenteral:ab OR parenteral:ti OR parenteral* OR 'parenteral nutrition' OR 'parenteral solution' OR 'parenteral nutrition'/exp) AND
2. (fish* OR fish:ti OR fish:ab OR 'fish oil'/exp OR omega* OR omega*:ti OR omega*:ab OR 'n 3':ti OR 'n 3':ab OR 'omega 3 fatty acid'/exp OR
3. lipid* OR lipid:ti OR lipid:ab OR 'lipid emulsion'/exp OR emulsion* OR emulsion:ti OR emulsion:ab * OR Immunonutrition:ti OR Immunonutrition:ab)) AND
4. (random* OR random*:ti OR random*:ab OR 'randomized controlled trial'/exp)

Web of science

The following string represents a draft Web of science search strategy (no limits[a]):

1. (TOPIC: (Fish oil) OR TOPIC: (Omega-3)) AND
2. TOPIC: (Parenteral nutrition)

[a]As suggested by the Cochrane recommendation on Systematic Reviews, no limits are imposed on the search and exclusion/inclusion criteria are checked manually on the hits.

Table S2. Summary of meta-analysis results for laboratory parameters.

**Outcome[a]**	**Studies (n)**	**Patients (n)**	**Effect measure (statistical method)**	**Effect estimate (95% CI; *p*-value)**
**AST (U/L)**	7	480	MD (IV, RE)	**–7.40 (–14.69 to –0.10; *p*=0.05)**
**ALT (U/L)**	10	962	MD (IV, RE)	**–9.31 (–18.44 to –0.17; *p*=0.05)**
**GGT (IU/L)**	5	398	MD (IV, RE)	**–8.74 (–24.91 to –2.66; *p*=0.02)**
Bilirubin (total; mg/dL)	7	838	MD (IV, RE)	0.03 (–1.35 to 1.41; *p*=0.97)
Bilirubin (direct; mg/dL)	3	157	MD (IV, RE)	–1.37 (–3.11 to 0.38; *p*=0.13)
Triglycerides (mg/dL)	6	754	MD (IV, RE)	–15.69 (–35.49 to 4.12; *p*=0.12)
**α-tocopherol (μmol/L)**	2	66	MD (IV, RE)	**15.25 (14.15 to 16.35; *p*<0.00001)**
**EPA[b]**	4	150	St.MD (IV, RE)	**2.98 (1.31 to 4.65; *p*<0.0005)**
**DHA[b]**	4	150	St.MD (IV, RE)	**1.27 (0.02 to2.53; *p*=0.05)**
AA[b]	4	150	St.MD (IV, RE)	0.14 (–0.38 to 0.65; *p*=0.61)
**LBT5 (pg/mL)**	2	63	MD (IV, RE)	**7.14 (5.59 to 8.68; *p*<0.00001)**
LBT4 (pg/mL)	2	63	MD (IV, RE)	–0.14 (–44.91 to 44.63; *p*=1.00)
**LBT5/LBT4**	3	126	MD (IV, FE)	**0.06 (0.05 to 0.07; *p*<0.00001)**
**TNF-α[b]**	11	994	St.MD (IV, RE)	**–0.49 (–0.81 to –0.16; *p*=0.003)**
CRP (mg/L)	10	1027	MD (IV, RE)	–10.19 (–20.56 to 0.18; *p*=0.05)
IL-6 (pg/mL)	11	974	MD (IV, RE)	–0.05 (–0.42 to 0.32; *p*=0.78)

Platelets (10^9/L)	3	416	MD (IV, FE)	1.12 (–13.41 to 15.66; *p*=0.88)
Creatinine (µmol/L)	6	505	MD (IV, FE)	–1.01 (–3.99 to 1.97; *p*=0.51)
Urea (mmol/L)	6	505	MD (IV, FE)	–0.13 (–0.36 to 0.10; *p*=0.26)

[a]Outcomes and effect estimates with significant difference marked as bold text.

[b]These laboratory parameters were expressed as heterogeneous measurement units, thus the standardised mean difference approach was adopted.

AA, arachidonic acid; ALT, alanine aminotransferase; AST, aspartate aminotransferase ; CI, confidence interval; CRP, C-reactive-protein; DHA, docosahexaenoic acid; EPA, eicosapentaenoic acid; FE, fixed effects; GGT, γ-glutamyl transferase; IL, interleukin; IV, inverse variance; LOS, length of stay; LT, leukotriene; MD, mean difference; M–H, Mantel-Haenszel; n, number; RE, random effects; RR, relative risk; St.MD, standardised mean difference; TNF, tumour necrosis factor.

Table S3. Comparison of clinical outcomes and cost-effectiveness results from previous meta-analyses and the current study.

**Clinical outcome**	**General hospitalised population Pradelli et al., 2020**[a]	**ICU population Pradelli et al., 2020**[b]	**Non-ICU hospitalised population (current study)**
Infections	RR 0.60 (95% CI 0.49–0.72; *p*<0.00001)<br>24 studies, 2154 patients	RR 0.62 (95% CI 0.45–0.86; *p*=0.004)<br>8 studies, 795 patients	RR 0.63 (95% CI 0.50–0.78; *p*<0.0001)<br>19 studies, 1690 patients
30-day mortality	RR 0.82 (95% CI 0.64–1.05; *p*=0.12)<br>20 studies, 1839 patients	RR 0.90 (95% CI 0.69–1.16; *p*=0.41)<br>12 studies, 925 patients	RR 0.46 (95% CI 0.20–1.08; *p*=0.07)<br>11 studies, 1246patients
Hospital LOS	–2.14 days (95% CI –1.36 to –2.93 days; *p*<0.00001)<br>26 studies, 2182 patients	–3.05 days (95% CI–1.07 to –5.03 days; *p*=0.003)<br>11 studies, 872 patients	–2.03 days (95% CI–1.23 to –2.84 days; *p*<0.00001)<br>18 studies, 1642 patients
Sepsis	RR 0.44 (95% CI 0.28–0.70; *p*<0.0004)<br>9 studies, 1141 patients	RR 0.56 (95% CI 0.26–1.19; *p*=0.13)<br>3 studies, 336 patients	RR 0.49 (95% CI 0.32–0.74; *p*=0.0009)<br>10 studies, 1117 patients
Cost-effectiveness/savings per patient in five European countries[c]	–	€3156–€5546	€1433–€2046

CI, confidence interval; LOS, length of stay; RR, relative risk.

[a]Pradelli L, Mayer K, Klek S, et al. ω-3 Fatty-acid enriched parenteral nutrition in hospitalized patients: systematic review with meta-analysis and trial sequential analysis. *JPEN J Parenter Enteral Nutr*. 2020;44(1):44–57.

[b]Pradelli L, Klek S, Mayer K, et al. Omega-3 fatty acid-containing parenteral nutrition in ICU patients: systematic review with meta-analysis and cost-effectiveness analysis. *Crit Care*. 2020;24(1):634.

[c]France, Germany, Italy, Spain and the UK.
